# Screening and Identification of Coastal Chilean Thraustochytrids for Arachidonic Acid Production: Biotechnological Potential of *Ulkenia visurgensis* Lng2-Strain

**DOI:** 10.3390/microorganisms11030559

**Published:** 2023-02-22

**Authors:** Cinthia Vasquez-Sandoval, José Navarrete, Paula Herrera-Herrera, Patricio Dantagnan, Paola Diaz-Navarrete, Patricia Arancibia-Avila, Claudia Oviedo

**Affiliations:** 1 Laboratorio de Bioprocesos y Biotratamientos, Departamento de Ingeniería en Maderas, Facultad de Ingeniería, Universidad del Bío-Bío, Concepción 4081112, Chile; 2 Departamento de Ciencia Agropecuarias y Acuícolas, Núcleo de Investigación en Producción Alimentaria y Facultad de Recursos Naturales, Universidad Católica de Temuco, Temuco 4810302, Chile; 3 Laboratorio de Ecofisiología y Microalgas, Departamento de Ciencias Básicas, Facultad de Ciencias, Universidad del Bío-Bío, Chillán 3800708, Chile; 4 Departamento de Química, Facultad de Ciencias, Universidad del Bío-Bío, Concepción 4081112, Chile

**Keywords:** thraustochytrids, arachidonic acid, *Ulkenia visurgesis*, very long chain unsaturated fatty acids, culture parameters

## Abstract

Thraustochytrids are unicellular heterotrophic marine protists that have been described as producing a high content of polyunsaturated fatty acids (PUFAs). Among them, arachidonic acid (ARA) stands out as a precursor of several mediators of pivotal importance for the immune system. However, the biotechnological potential of thraustochytrids for ARA production has not been developed. The objective of this study is to isolate and identify native strains from different Chilean coastal environments and evaluate in vitro the effect of culture parameters such as C/N ratio (19 and 33) and temperature (15 °C and 23 °C) on biomass production and arachidonic acid content. A total of nine strains were identified and classified into four genera of the Thraustochitridae family. The Lng2 strain with 99% identity belongs to the species *Ulkenia visurgenis* and was the most prominent one for ARA production. Temperature had an effect on the PUFA profile but not on the ARA content nor on the biomass yield. Additionally, the C/N ratio has been identified as a key parameter. The ARA productivity increased by 92% (from 0.6 to 8.3 ARA mg/g-DW) and its total biomass by 62.7% (from 1.9 to 5.1 g/L) at a high C/N ratio (33) as compared to the control.

## 1. Introduction

Thraustochytrids are unicellular heterotrophic marine protists widely distributed in marine and estuarine ecosystems and frequently associated with organic material in decay [1,2]. Currently, there are nine genera of the family Thraustochytriidae: *Thraustochytrium, Japonochytrium Schizochytrium, Ulkenia, Aurantiochytrium, Sicyoidochytrium, Parietichytrium, Botryochytrium*, and *Monorhizochytrium* and they are classified according to their morphological characteristics, ultrastructure, life cycles, and biochemical markers [1]. These microorganisms have been characterized as oleaginous since they use two independent routes for fatty acid synthesis: A standard pathway (aerobic) for the production of saturated fatty acids; and a polyketide synthase-like synthase pathway (anaerobic) to synthesize the very long chain unsaturated fatty acids PUFAs (VLCPUFAs) such as DHA (22:6n-3) and arachidonic acid (ARA) (20:4n-6) [1,3,4,5,6,7,8,9,10].

PUFAs are necessary as nutrients for human and animal health, growth, and development [11,12]. Two families represent these PUFAs: n-6 (u ω-6) and n-3 (u ω-3), which are biosynthesized from linoleic acid and linolenic acid, respectively; humans cannot synthesize the latter; therefore, they must be incorporated through diet [11,12,13,14]. Among the ω-6 series, ARA stands out as one of the cell membrane phospholipids, esterified in the Sn-2 position. Free ARA, catalyzed by a phospholipase, is a precursor of various physiological mediators involved in inflammatory responses such as prostaglandins and thromboxanes [15].

ARA and DHA are fundamental in the formation of the structural genesis and functionality of the human nervous and visual systems and act as neuroprotective agents against degenerative diseases such as Parkinson’s and Alzheimer’s [16,17,18,19]. Both fatty acids constitute more than 30% of the lipid structure of the brain and of the retinal rods and cones [11]. In addition, ARA regulates electrical activity in the brain, muscles, and heart by acting on voltage-gated ion channels [11,14]. The FAO recommends an additional intake of ARA and DHA in premature infants, without breastfeeding, to improve the growth and development of the central nervous system and retina [11,12]. Poor intake of ARA can cause hair loss, fatty liver, anemia, and reduced fertility in adults [11,13]. In fish feed, studies reveal important and diverse roles of ARA in various physiological and nutritional processes [20,21,22]. For example, the inclusion of ARA and vitamin E in the diet of the Atlantic salmon (*Salmo salar)* can improve the indicators of nonspecific immunity, leucocyte respiratory burst, lysozyme activity, and reduction of cumulative mortality [23].

Initially, some oleaginous plants were considered as sustainable sources in the production of PUFAs, especially the linoleic precursor. However, their use is limited, since genetic engineering is necessary to obtain modified plants that produce fatty acids in commercial quantities [24]. Microorganisms have also been described for PUFAs production such as bacteria, filamentous fungi, microalgae, and heterotrophic protists [25,26,27,28].

In the *Mortierella alpina* fungus, the ARA is 80% with respect to the total lipids, but it requires long culture periods, i.e., two weeks, to reach this value [29,30,31]. Bacterial strains *Shwenella* sp. and *Colwellia* sp. have also been described as producing DHA and EPA. However, they are not considered viable for commercial production since they have a lower accumulation of PUFAs with respect to total lipids (2–5%) [27,32]. Other candidates are autotrophic microalgae. *Parietochloris incisa* and *Porphyridium purpureum* stand out, the latter, red unicellular microalgae, under stress conditions (of light intensity, pH, temperature, salinity, and nutrient deficiency) can reach 40% ARA of total fatty acids [33,34]. However, its large-scale commercialization is limited by its autotrophic metabolism, which implies low accessibility of cells to light when there is high cell density, evaporation problems, and the use of large extensions of land for its cultivation [35,36].

Reports on ARA abundance with respect to their total fatty acid profile are limited for heterotrophic protists. There are data available on *Schizochytrium* sp. and *Crypthecodinium cohnii* (*C. cohnii* ATCC 50060), both with 2.2% as can be inferred from Ganuza [37]. In the *Ulkenia* sp. KF13 strain, it is 1.7% [38] and in *Thraustochytriidae* sp. AS4-A1, it is 6.2% [39]. However, these microorganisms have the natural ability to accumulate a high content of lipids, which can be increased by optimizing key factors in culture [40,41,42,43,44]. In this context, and in an effort aimed at identifying new sources of ARA, heterotrophic microorganisms of the Thraustochytriaceae family are becoming relevant. 

Nutritional and environmental conditions have an important effect on the total lipid content and on its PUFA fraction in thraustochytrids. An important role of parameters such as C/N ratio and temperature has been described [43,44,45,46,47]. Indeed, N deficiency in the culture medium would inhibit cell division; hence, the C flow is directed towards lipid production [43]. On the other hand, temperature affects cell growth and PUFA composition of most microorganisms [45,46]. Hence, under low temperature conditions, more ARA and DHA are synthesized and incorporated into the membrane to maintain its fluidity and permeability [43,44,45].

Considering the biotechnological potential of thraustochytridiae in the production of ARA, studies that undertake the search for ARA-producing strains are needed. Accordingly, the objective of this work was to isolate and identify thraustochytrids from different Chilean estuarine and coastal environments, evaluating in vitro their ARA production and studying the effect of culture parameters such as C/N ratio and temperature on biomass production and ARA content.

## 2. Materials and Methods

### 2.1. Isolation and Culture Conditions

**Figure 1:**
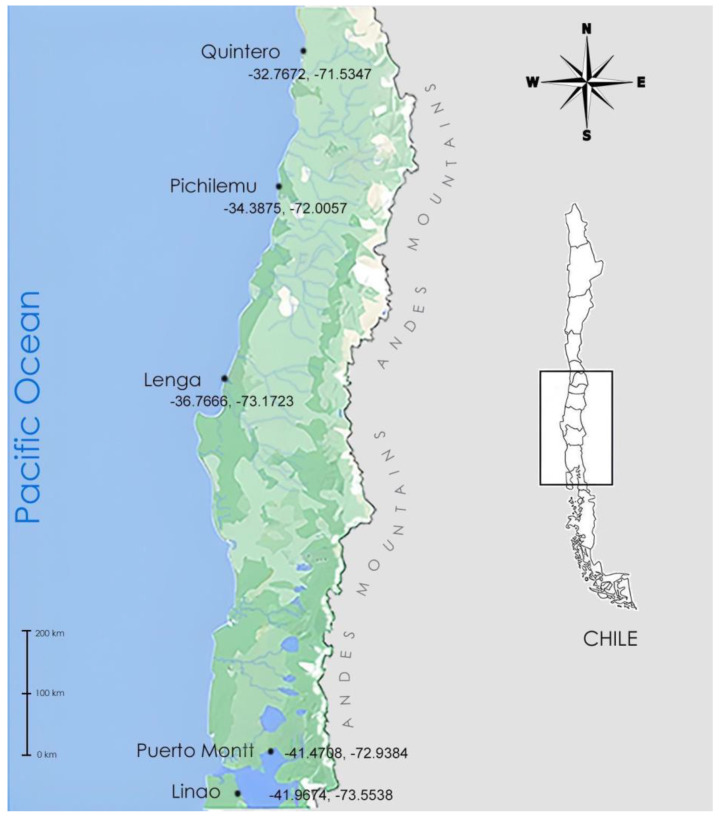
Sampling sites. Map of diverse sampling sites (with GPS coordinates) at the Chilean coast for thraustochytrids isolation.

Marine samples were collected from five different Chilean coastal sites and habitats. Samples were obtained from the water column (15–20 cm depth) or sediment. A sampling sites map was constructed using the Google Maps platform (Figure 1). For thraustochytrids isolation, the pollen technique was performed [48]. The presence of thraustochytrids adhered to pine pollen was verified by optical microscopy. Subsequently, samples were filtered (nylon filter, 20 μm) and cultivated on solid culture medium 790 (yeast extract 1 g/L, peptone 1 g/L, glucose 5 g/L, and agar 15 g/L) prepared in diluted artificial seawater (NaCl 27.12 g/L, MgCl_2_ × 6H_2_O 5.23 g/L, MgSO_4_ × 7H_2_O 6.77 g/L, CaCl_2_ × H_2_O 0.15 g/L, KCl 0.725 g/L, and NaHCO_3_ 0.202 g/L) and a final concentration of 0.3 g/L of streptomycin and penicillin G (Sigma Aldrich Co., Steinheim, Germany). Incubation was performed at 22 °C for 5 to 7 days. Selected colonies were subcultured in liquid 790. 

### 2.2. DNA Extraction and PCR Assays

Freeze-dried biomass from pure cultures was resuspended with a lysis buffer (200 μL; 0.25 M Tris-Cl; 0.1 M Na2-EDTA; 2% *w/v* SDS; 0.1 M NaCl at pH 8.2). DNA was extracted with phenol/chloroform/isoamyl alcohol cold (4 °C) and precipitated with cold ethanol (70%, 4 °C). For molecular identification, partial regions of the 18S rRNA gene were amplified by PCR using a master mix reaction (Buffer 5 µL; dNTPs 1 µL; MgCL_2_ (50 mM) 1.5 µL; ADN 5 µL; Taq 0.2 µL) and two pairs of specific primers [49,50,51] (18S001′5 AACCTGGTTGATCCTGCCAGTA 3′, 18S13′ 5CCTTGTTACGACTTCACCTTCCTCT 3′ and FA2′ 5 GTCTGGTGCCAGCAGCCGCG 3′, RA3 CAATCGGTAGGTGCGACGGGCGG). The cyclic amplification program was: 3 min denaturation at 95 °C, 35 cycles of 1 min amplification at 94 °C, 1 min at 50 °C, and 1 min at 72 °C, with 10 min extension for the elongation stage. The PCR products were visualized on a 2% green agarose gel.

The obtained sequences were compared with those available in the Genbank database (National Center for Biotechnology Information, USA: NCBI Home page http://www.ncbi accessed on 10 October 2021). The search for the most related homologous sequences was performed using the BLAST program available on the NCBI web server.

Taxonomic evaluation, DNA alignment, and interpretation of sequences were performed by using Geneious Prime software [52]. BLAST was performed against the GenBank database. Representative sequences alignment was done by using MUSCLE [53]. Jmodeltest 2.1.10 [54] software was used for the alignment best-fit model of nucleotide substitution selection. Phylogenetic reconstruction was done by using Bayesian inference (BI) method in MrBayes 3.2.6 [55] geneious plugin.

### 2.3. Screening for High ARA-Producing Strains and Total Biomass Assessment

Pure strains obtained in 2.2 were cultivated in triplicate, in 100 mL 790-liquid medium using 250 mL flasks, at 20 °C and 120 rpm for 7 days. To determine the final biomass resulting from each cultivated strain, the biomass was washed thrice, centrifuged at 1798 rcf for 5 min, and finally, the biomass was lyophilized, and dry weight (DW) was determined gravimetrically.

For the extraction of total lipids, Folch’s method [56] was applied. Each sample was homogenized in a chloroform/methanol (2:1, *v/v*) mixture, then centrifuged at 1614 rcf in a refrigerated centrifuge (Ependorf, model 4303), to separate the water-soluble and organic phases. The organic phase was then separated, and the chloroform was evaporated using a mini-evaporator. The amount of total lipids present in each sample was expressed as a percentage of its dry weight. Fatty acids methyl esters (FAME) were prepared from the extracted lipids according to Morrison and Smith (1964) [57].

Fatty acids were separated in a gas chromatograph (Hewlett Packard 6890 series II Plus, Wilmington, NC, USA) using a 30 m × 0.25 mm × 0.20 µm capillary column SP^TM^ 2380 (Supelco, Bellefonte, PA, USA). Helium was used as a carrier gas. FAME was analyzed by comparison with a well characterized standard such as SUPELCO^TM^ 37 component FAME Mix (Sigma-Aldrich, St. Louis, MO, USA). Fatty acids were expressed as percentage of total identified FAME. The productivity of ARA, EPA, and DHA were expressed in mg/dry biomass. 

### 2.4. Temperature Effect on Growth Curve and Glucose Consumption 

The highest ARA-producing strains detected in 2.3, i.e., ARA mg /biomass g-DW were cultivated at different temperatures (15 °C and 23 °C) for 5.5 days at 120 rpm. Biomass was estimated in triplicate, initially every 3 h and then every 12 h, by cell DW as follows: Cell cultures were harvested, centrifuged (1798 rcf for 5 min), washed (three times) with distilled water, and subsequently lyophilized for gravimetric determination. Glucose consumption was estimated spectrophotometrically at the same time intervals as biomass, using a commercial SPINREACT Kit (Glucose-LQ).

### 2.5. C/N Ratio Effect on Productivity de ARA 

To evaluate the effect of C/N ratio on ARA production, batch fermentations were performed using the selected *Ulkenia visurgensis* (Leng2 strain).

An accurate determination of C and N content present in the reagents used was performed by elemental analysis Thermo Scientific (FlashSmart™ Elemental Analyzer) at Universidad Católica de Temuco (Table 1).

While preparing the culture media, the C/N ratio was modified by altering the content of the main carbon source (glucose from 10 to 40 g/L) and the content of Peptone (from 2 to 4 g/L) and yeast extract (from 1 to 2 g/L).

The C/N ratio of each medium, control (15), C/N 19 (1), and C/N 33 (2), was calculated considering the amount of carbon and nitrogen provided by the reagents: glucose, peptone, and yeast extract. The experiments were performed in triplicate and cultured for 5 days with orbital agitation (120 rpm) at 23 °C.

The C/N ratio of each medium, listed in Table 1, was calculated by the amount of carbon and nitrogen provided by glucose, peptone, and yeast extract. The experiments were performed in triplicate and cultured for 5 days with orbital agitation (120 rpm) at 23 °C.

### 2.6. Statistical Analysis

Statistical analyses (ANOVA and Student’s *t*-test) were performed using the MATLAB program (R2019b, The MathWorks Inc., Natick, MA, USA; 2018) to compare biomass and ARA yield obtained in the native strains and the treatments with C/N ratio in the Lng2 strain, respectively. A significance level of 95% (*p* < 0.05) was used to determine significant differences.

## 3. Results

### 3.1. Morphological Characteristics and Genetic Identification

Using the pine pollen technique, nine strains (Table 2) were obtained from diverse Chilean coastal environments (Figure 1). Through the partial amplification of the 18S rRNA gene, it was possible to identify four genera of thraustochytrids: *Ulkenia* sp: strains Quint1, LNO, and PtoM (GenBank accession numbers|AB810968.1|), *U. visurgensis*; strain Lng2 (GenBank accession numbers|HQ228958.1|), *Thraustochytrium* sp.; strains Lng1, Lng3, Pich3 and Pich4 (GenBank accession numbers|HQ228958.1|), and *Botryochytrium* sp.; strain Lng6 (GenBank accession numbers|AB973506.1|) (Table 2). All strains exhibit a high identity over 99% with the correspondent sequences stored in GenBank, thus confirming the taxonomic classification within the Thraustrochytriidae Family.

**Figure 2:**
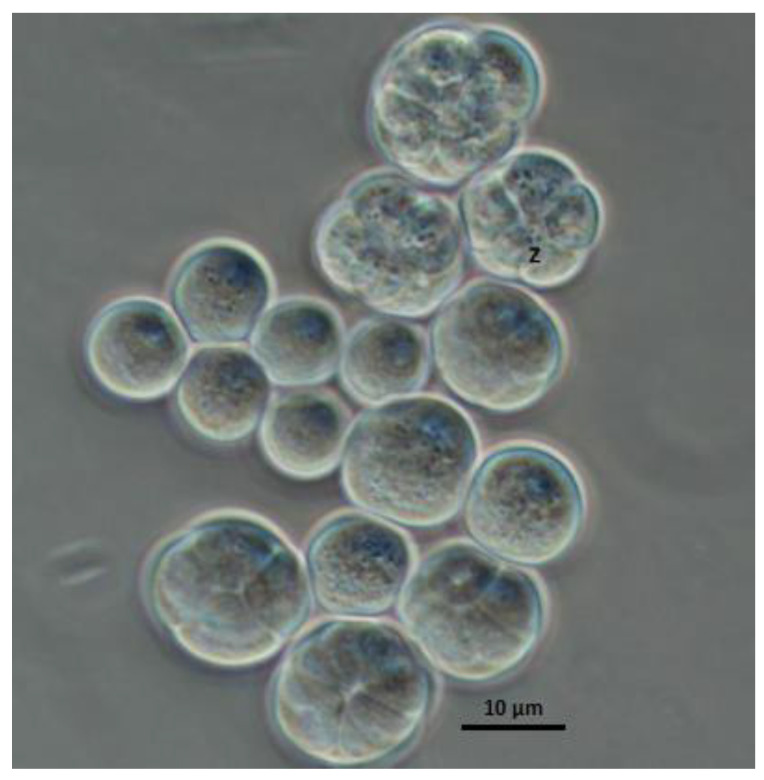
Light microscopy image of strain Lng2 isolated in Lenga, Chile. Mature sporangium with zoospores (z). Scale bar 1000×.

**Figure 3:**
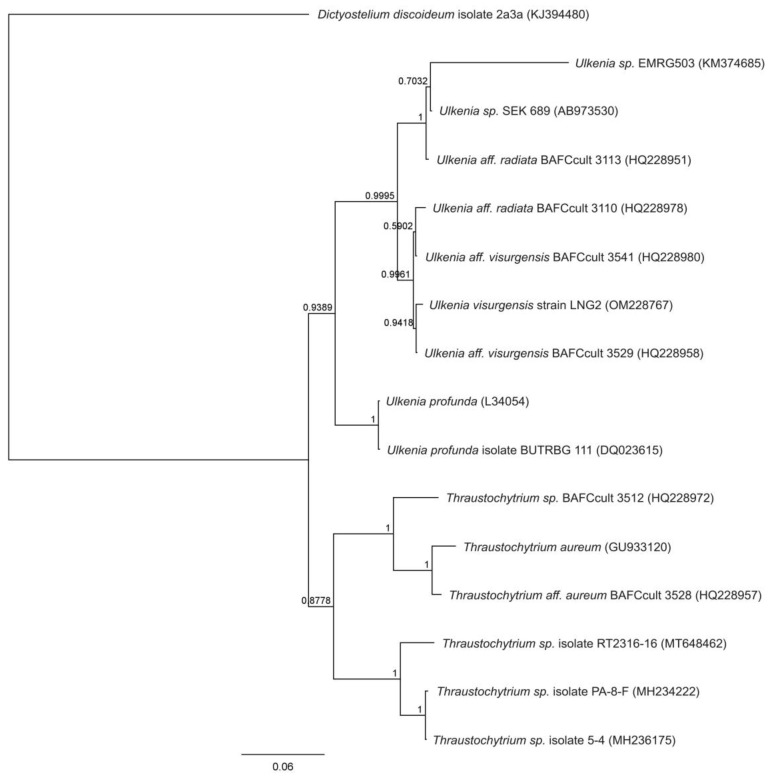
Phylogenetic tree of strain Lng2, based in molecular analysis on complete alignment of the 18S rRNA gen.

Under microscopic observation, the strain Lng2 (*U. visurgensis*) presents a spherical shape with mature sporangia containing zoospores (Figure 2). The comparison of the 18S rRNA gene shows that the Lng2 strain exhibits a common ancestor, with two strains; *Ulkenia aff visurgensis* BAFCult 3541 (HQ228980) and *Ulkenia aff visurgensis* BAFCult 3529 (HQ228958), with a bootstrap value of 0.99 and 0.94, respectively (Figure 3). The 18S rRNA partial sequence of strain Lng2 are available in GenBank (accession number: OM228767) (Figure 3).

#### 3.1.1. Screening for High ARA-Producing Strains

The productivity of ARA, EPA, and DHA in mg/biomass g-DW and their respective percentages (related to their total lipids) was determined in all the native strains isolates (Table 3). ARA was detected in the nine identified strains with percentages of total lipids from a minimum of 2.59% to a maximum of 9.31% in the Pch3 and LNO strains, respectively. The maximum cellular biomass (DW) corresponds to the Lng3 strain with 1.12 g-DW/L. However, its percentage of ARA (2.97%) is low, limiting its biotechnological potential. On the other hand, the Lng2 strain isolated from an estuarine zone, with 5.44% of ARA has the highest productivity (5.81 ARA mg/ biomass g DW); although its biomass is low (0.45 g/L), its DHA percentage levels (44.78%), EPA (16.26%), and total lipids (10.68%) are remarkably high compared to the other strains. Based on Lng2 (*U. visurgensis*) high PUFAs productivity including ARA, the culture parameters were assayed for that strain by evaluating the effect of temperature and the C/N ratio on the productivity of ARA and cell biomass.

**Figure 4:**
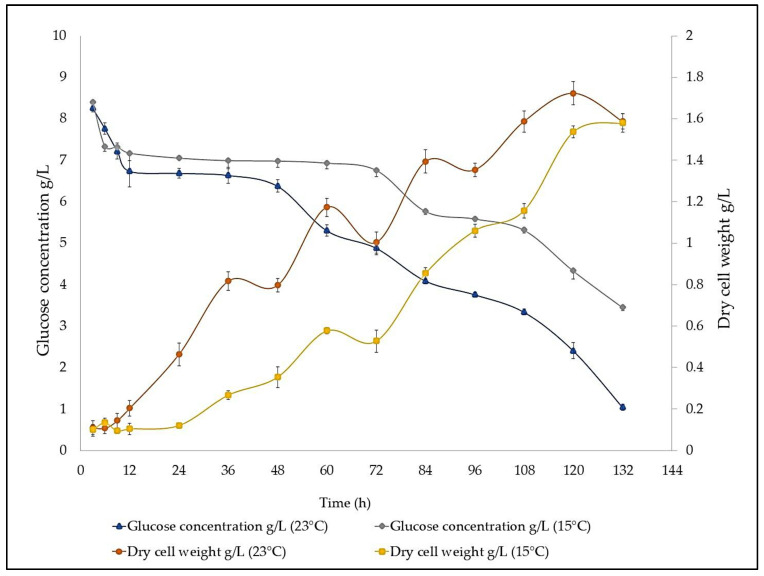
Growth curve and glucose consumption at 23 °C and 15 °C in Lng2 strain (*U. visurgensis*). The bars indicate ± standard error (n = 3).

Figure 4 shows the total biomass growth kinetics of Lng2 strain during a 5.5 d (132 h) culture at two temperatures (15 and 23 °C) and its respective glucose consumption. Lng2 grew faster at 23 °C than at 15 °C. The exponential growth 3–5 d (72–120 h) and the stationary phase (from 5 d onwards) occurred at the same intervals for both temperatures. At 23 °C, the maximum biomass was obtained at 1.723 g-DW/L at 120 h. Additionally, in relation to glucose, it was observed that the remaining substrate is 1.04 g glucose/L at 23 °C (132 h). However, at 15 °C and similar culture hours, the value is three times higher than in the higher temperature (3.44 g glucose/L), which is an interesting trait for industrial scaling prospects.

The effect of temperature (15 °C and 23 °C) on the fatty acid profile and yield of ARA was evaluated during the exponential phase (from 3 d) and at the beginning of the stationary phase (5 d) (Table 4). The maximum biomass is 1.46 g-DW/L (exponential phase) and the minimum is 1.09 g-DW/L (stationary phase), both at 23 °C. The maximum yield of ARA (mg/biomass g DW) is at 3.87 (15 °C) and the minimum is at 1.88 (23 °C), both in the exponential phase.

Lower temperature does affect the profile of fatty acids, mainly PUFAs. At 15 °C, the total percentage of ARA in the biomass is 47.1% higher than the culture at 23 °C (3.99 and 1.88%, respectively) during the exponential phase (Table 4). In addition, PUFAs represent 44% of the total fatty acids at the same temperature and cultivated period.

#### 3.1.2. Evaluation of C/N Ratio on the Productivity of ARA 

**Figure 5:**
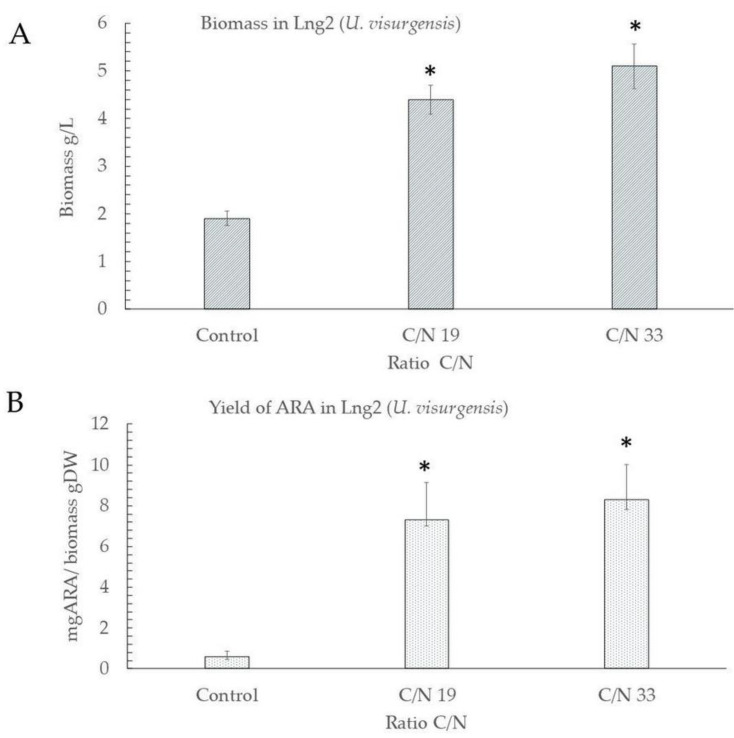
Effect of different C/N treatments on cell biomass (**A**) and ARA yields in Lng2 (*U. visurgensis*) strain (**B**). The bars indicate ± standard error (n = 3). * Indicates significant differences between control and C/N treatments (*p* < 0.05).

Figure 5 describes the important effect that the C/N ratio has on cell biomass and ARA productivity. At a C/N ratio of 19, the biomass increases from 1.9 g-DW /L (control) to 4.4 g-DW/L (i.e., a 56.8% increase). However, this value is even higher at a C/N ratio of 33 with final biomass of 5.3 g-DW/L, i.e., 64.1% more than the control. Additionally, the highest productivity of ARA also corresponds to this last C/N ratio of 33 with 8.3 ARA mg/biomass g-DW, which is the highest ARA yield found in this study.

**Figure 6:**
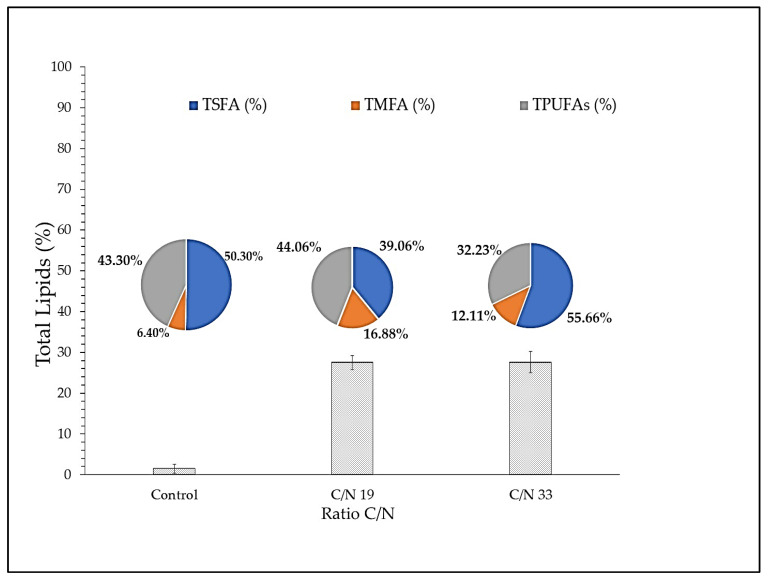
C/N ratio effect on Lng2 (*U. visurgensis*) strain; cultivated at 23 °C, for 5 d. Bar chart: Effect on the total lipid content (% calculated over dry weight biomass). Pie chart: Effect on the lipid profile (% of total lipids). TSFA: total saturated fatty acids, TMFA: total monosaturated fatty acids and TPUFA: total polyunsaturated fatty acids. The bars indicate + standard error (n = 3).

Figure 6 shows the distribution of fatty acids with respect to the carbon and nitrogen ratio present in the culture medium. Saturated fatty acids predominate in the C/N ratios of 19 and 33 with percentages of 50.3% and 55.6%, respectively. Additionally, PUFAs are the second most important group of total lipids, their values are 44.1% for the control strain and 43.1% at C/N ratio of 19 and 32.2 for the C/N ratio of 33. The C/N ratio has an important effect on the total lipids (%). This value increase from 1.5% to 27.5% and 27.6% in a C/N ratio of 19 and 33, respectively, was achieved, i.e., 94.6% more than the control. Although an increase of 1.7-fold of the C/N ratio does not affect the proportion of total lipids, it triggers a high ARA yield (Figure 5B).

#### 3.1.3. Effect of Culture Conditions on Cell Biomass and ARA Yields

Among all the identified native strains, non-significant differences between their total biomass yields were found. The same trend was observed for the ARA yields among these native strains, where non-significant differences were found (ANOVA, *p* > 0.05) (Table 3).

Temperature had a non-significant effect on the LNG2 strain cultures (Table 4. ANOVA, *p* > 0.05), whereas the two C/N ratios assayed had a significant effect as compared to the control, on the LNG2 strain cultures (Figure 5 Student’s test, *p* < 0.05).

## 4. Discussion

### 4.1. Morphological Characteristics and Genetic Identification

The literature describes a wide geographical distribution of thraustochytrids and highlights their versatility to adapt to various marine environments with particular characteristics of temperature, light, and suspended decomposing material [1,3,38]. In this study, samples of thraustochytrids were isolated from coastal environments along Chile. The genus *Ulkenia* sp. was the most frequently identified. Thraustochytrids have been reported in mangroves [58,59] whose intertidal zones are exposed to freshwater courses. Interestingly, three genera of thraustochytrids (*Thraustochytrium* sp., *U. visurgensis*, and *Botryochytrium* sp.) were identified in the Lenga estuary, whose salinity fluctuates due to freshwater inflows from a river. Additionally, to our knowledge, this research reports the genus *Botryochytrium* sp. for the first time in Chile [6,7,39,60]. 

### 4.2. Biomass and Yield in Strain Natives 

The Lng2 native strain of the genus *U. visugensis* has a 5.44% ARA content out of the total lipids and its yield is 5.81 ARA mg/ biomass g-DW (Table 2). These values are significant as compared to the ones reported of strains isolated from an estuary and cultivated in an optimized medium (composed of 1 g peptone, 1 g yeast extract, 20 g glucose, 1 L seawater, 25 °C) where the yields for *S. mangrovei* strains I AO-1 and IXm-6, *Schizochitryum* sp. strain BSn-1 and *Thraustrochytrium* sp. strain Ira-8 are 0.9, 0.4, 0.7, and 5.5 ARA mg/biomass g-DW, respectively [61]. The latter reported yields are lower than the ones obtained from the native Lng2 strain cultivated in a non-optimized medium in this research. 

### 4.3. Evaluation of the Effect of Temperature on Strain Lng2 (U.visurgensis)

Temperature is one of the most important environmental factors affecting growth rate. Low temperatures inhibit cell development but stimulate the synthesis of fatty acids, mainly PUFAs [28,45]. For example, the biomass in *Aurantiochytrium* sp. strain mh0186 grown at 10 °C (0.6 g-DW/L) was considerably lower than at 15 °C (6.7 g-DW/L). However, the percentage of DHA increased from 59% to 75% (total lipids basis) when the temperature dropped from 15 °C to 10 °C [62]. A proteomics study indicates that cold stress inhibits the supply of cellular energy by glycolysis and the Krebs cycle, which could explain the low biomass yields [63]. However, in this study, a greater temperature difference (23 °C and 15 °C) did not significantly affect the cell biomass yield of Lng2 strain (always over 1 g-DW/L) (Table 4). In general, this yield fits in the usual range reported for batch cultures in thraustochytrids (from 0.5 to 2.3 g-DW/L) [6]. A feasible explanation for the non-significant effect of temperature on biomass yields found in the Lng2 strain could be related to the estuarine origin of that strain (Table 2). As an estuary is a dynamic ecosystem, its marine population is constantly subjected to physicochemical changes [64]. These adaptability traits [65,66] could account for the behavior of the Lng2 strain (isolated from an estuary) whose biomass yield is not affected by the assayed temperatures. Therefore, similar biomass yields are obtained regardless of whether it is cultivated at 15 °C or 23 °C (Table 4). On the other hand, in the Lng2 strain, low temperatures stimulate the synthesis of PUFAS and increase its ARA content as well, with differences of 23% (from 15 °C to 23 °C) and 53% (from 15 °C to 23 °C), respectively, at the exponential phase. These results are in accordance with previous reports [62,67,68]. These changes are associated with membrane integrity and functional maintenance. ARA and DHA are incorporated into the membrane to preserve its fluidity and permeability [11,45,46]. On the other hand, the yield of ARA is similar at both culture temperatures assayed (Table 4). The plasticity of the strain to adapt to low temperatures without slowing down its cell biomass yield hints at the potential of the Lng2 strain for both ARA and PUFAs production in industrial-scale fermentation.

### 4.4. Evaluation the Reason C/N in the Biomass and Yield of ARA in Strain Lng2 (U. visurgensis) 

Nutrients such as carbon and nitrogen are key in the cellular development of thraustochytrids. Our results confirm that the concentration of glucose (from 5 g-DW/L to 40 g-DW/L) as a carbon source directly affects the increase in its total biomass (i.e., 62.7% with respect to the control). Previous studies [7] with the strain *T. areum* ATCC 34304 obtained an increase of 13.8% when glucose was added to the culture medium (from 5 to 20 g-DW/L). Accordingly, the yield of ARA had a significant increase at a higher concentration of glucose in culture reaching up to 8.3 ARA mg/biomass g-DW, i.e., 92.8% with respect to the control in just 3 days. Other studies on the microalga *Porphyridium purpureum* obtained yields of 10.87 and 10.61 ARA mg/biomass g-DW after 18 days of culture, by adding NaCO3 and 5-ALA, respectively, hence obtaining an increase of 11% and 16.1% with respect to the control conditions [69].

The results obtained in this study indicate that the C/N ratio has a significant effect on ARA yield and final biomass. However, excess carbon leads to the synthesis of saturated lipids and to an inhibition of the synthesis of membrane-associated PUFAs [70]. Similar results were observed in this research at a high C/N ratio (33) as the percentage of saturated fatty acids was 9.5% higher than the control and 25.6% lower in PUFAs content. However, there was an increase in total lipids of 94.5% with respect to control conditions (Figure 6). It is necessary in future studies to accurately determine the N concentration required by the Lng2 strain in order to optimize ARA synthesis.

Thraustochytrids are fast-growing heterotrophic organisms that can use alternative and low-cost carbon sources for cultivation, for example, lignocellulosic biomass [47] and waste from pig industries [71]. This nutritional plasticity allows efficient use of resources and decreases the costs associated with production, and desired characteristics in a strain with commercial potential [72,73,74,75]. The increase in ARA productivity in the Lng2 *U. visurgensis* strain mediated by nutritional variation is a key factor to be considered for future studies by optimizing the culture medium. In all, the results found in this study highlight the Lng2 strain as a potential candidate for ARA production.

## 5. Conclusions

A total of nine strains collected in diverse coastal Chilean environments were isolated and successfully maintained in laboratory conditions. They were classified into four genera corresponding to the thraustochitridae family; *Thraustochytrium* sp., *U. visurgensis*, *Botryochytrium* sp., and *Ulkenia* sp.

The Lng2 strain was identified as *U. visurgensis* with a 99.43% identity. Their sequences were included in the GenBank database (number access: OM228767.1). An initial screening singled out the Lng2 strain (*U. visurgensis*) as the most promising one for ARA production. Results derived from the temperature effect (15 °C and 23 °C) indicate that this factor affects the PUFA profile but not the ARA content nor the biomass yield. 

The proportion of nutrients (carbon and nitrogen) is a key parameter for the Lng2 strain. The productivity of ARA increased by 92% (from 0.6 to 8.3 ARA mg/g-DW) and its total biomass by 62.7% (from 1.9 to 5.1 g/L) at a high C/N ratio (33) as compared to control. This research provides evidence of the potential of Lng2 strain for ARA production.

## Figures and Tables

**Table 1:** Carbon and nitrogen content of media used.

Reagents	Total Organic Carbon TOC (%)	Total N (%)
Glucose (Gibco)	40	-
Peptone Bacto^TM^ (Gibco)	41	10
Yeast extract (Gibco)	25.35	7.22

**Table 2:** Thraustochytrid strains isolated and identified in this study from different Chilean coastal environments.

Sampling Site	Sampling Date	Isolation	Number of Strains	Genus Level Classification
Quintero (Quint1)	January 2020	Water column	1	*Ulkenia* sp.
Pichilemu (PiCh3-PiCh4)	September 2019	Water column	2	*Thraustochytrium* sp.
Lenga Estuario (Lng 1-Lng2-Lng3-Lng6)	April 2019	Water column	4	*Thraustochytrium* sp. *Ulkenia visurgensis* *Botryochytrium* sp.
Puerto Montt (PtoM)	April 2021	Water column	1	*Ulkenia* sp.
Linao (LNO)	April 2021	Marine sediment	1	*Ulkenia* sp.

**Table 3:** Total yield of biomass, total lipids, and specific productivity of ARA, EPA, and DHA found in Chilean native thraustochytrid strains isolated in this study.

Strain	Pich3	Pich4	Quint1	Lng1	Lng2	Lng3	Lng6	LNO	PtoM
**Biomass (g-DW/L)**	**0.35 ± 0.25**	**0.54 + 0.03**	**0.55 + 0.80**	**0.55 ± 0.67**	**0.45 ± 0.13**	**1.12 ± 0.05**	**0.72 ± 0.34**	**0.11 ± 0.01**	**0.5 ± 0.17**
* Total Lipids (%)	8.35 ± 1.62	9.14 ± 0.99	7.16 ± 2.54	10.66 ± 0.75	10.68 ± 0.98	16.35 ± 0.89	4.88 ± 2.96	3.89 ± 3.63	6.29 ± 0.93
ARA ^1^(%)	2.59 ± 0.77	3.11 ± 0.97	5.13 ± 0.17	4.25 ± 1.42	5.44 ± 0.75	2.97 ± 1.89	8.24 ± 2.35	9.31 ± 1.28	4.46 ± 0.65
EPA ^1^ (%)	11.66 ± 1.20	12.1 ± 0.87	4.46 ± 0.32	8.66 ± 0.86	16.26 ± 1.17	6.03 ± 0.49	15.17 ± 0.57	11.76 ± 2.18	3.63 ± 0.19
DHA ^1^(%)	57.01 ± 1.18	58.23 ± 1.15	17.8 + 0.96	31.46 ± 0.73	44.78 ± 0.84	22.16 ± 0.91	28.42 ± 2.99	32.58 ± 2.01	14.28 ± 0.55
**ARA mg/biomass g-DW**	**2.16 ± 0.89**	**2.84 ± 1.02**	**3.67 ± 0.62**	**4.53 ± 0.90**	**5.81 ± 1.17**	**4.86 ± 0.95**	**4.02 ± 0.76**	**3.62 ± 0.91**	**2.8 ± 2.01**
EPA mg/biomass g DW	9.74 ± 1.07	11.06 ± 0.75	3.19 ± 0.87	9.23 ± 0.56	17.37 ± 0.95	9.86 ± 1.02	7.4 ± 0.89	4.57 ± 0.57	2.28 ± 0.83
DHA mg/biomass g DW	47.61 ± 1.08	53.22 ± 2.91	12.74 ± 1.19	33.54 ± 0.03	47.83 ± 0.05	36.23 ± 0.82	13.87 ± 0.23	12.66 ± 0.76	8.98 ± 0.79

* Total lipids: (%) of dry weight biomass. ^1^ ARA, EPA, DHA: abundance with respect to total lipids (%). ±Mean values and their respective standard deviations are presented, n = 3. Effect of temperature: Growth curve and glucose consumption at 23 °C and 15 °C.

**Table 4:** Fatty acid profile of Lng2 strain (*U. visurgensis*) cultivated at two temperatures at its exponential (72 h) and stationary (120 h) phase.

Strain LNG2	15 °C			23 °C
	72 h	120 h	72 h	120 h
Biomass g-DW/L	1.14 ± 0.23	1.24 ± 0.11	1.46 ± 0.45	1.09 ± 0.25
^1^ Total Lipids (%)	8.52 ± 1.05	9.65 ± 1.79	7.79 ± 0.98	8.99 ± 1.45
^1^ C12:0	0.16 ± 0.04	0.21 ± 0.05	0.3 ± 0.17	0.28 ± 0.13
^1^ C14:0	1.58 ± 0.86	1.92 ± 1.26	2.29 ± 0.60	1.98 ± 0.09
^1^ C15:0	1.10 ± 0.19	1.57 ± 0.36	1.45 ± 0.42	1.37 ± 0.37
^1^ C16:0	33.79 ± 1.09	39.98 ± 4.28	43.36 ± 3.79	38.78 ± 4.62
^1^ C17:0	0.61 ± 0.23	1.04 ± 0.44	0.76 ± 0.27	0.77 ± 0.34
^1^ C18:0	6.23 ± 1.09	8.40 ± 1.46	7.76 ± 3.15	7.86 ± 3.65
^1^ C18:3n3	0.03 ± 0.03	0.03 ± 0.02	0 ± 0.00	0.03 ± 0.02
^1^ C20:2	0.09 ± 0.04	0.11 ± 0.01	0.07 ± 0.06	0.1 ± 0.02
^1^ C20:4n6 ARA	3.99 ± 1.49	3.10 ± 1.59	1.88 ± 1.12	3.07 ± 1.07
^1^ C20:5n3	4.67 ± 2.82	3.34 ± 1.76	4.44 ± 2.86	4.81 ± 3.17
^1^ C22:6n3	33.65 ± 0.89	26.97 ± 1.10	21.81 ± 0.10	25.09 ± 2.14
^2^ ARA mg/biomass g-DW	3.40 ± 0.89	2.99 ± 1.00	1.46 ± 0.98	2.79 ± 0.40
^1^ Total PUFAs	44.00 ± 3.53	29.48 ± 4.21	34.87 ± 0.30	34.45 ± 3.86
^1^ Total SFAs	45.38 ± 2.29	56.87 ± 1.83	54.56 ± 7.48	52.27 ± 4.30
^1^ Total MFAs	10.63 ± 1.80	13.65 ± 1.97	10.56 ± 2.01	13.28 ± 3.14

Note: ± Mean values and their respective standard deviations are presented, n = 3. ^1^ Indicator of Fatty acids expressed as percentage of total identified FAME. ^2^ The productivity of ARA was expressed in mg/dry biomass.

## Data Availability

Partial 18S gene sequencing strain Lng2 GenBank (accession number: OM228767).
